# The Effect of Looming and Receding Sounds on the Perceived In-Depth Orientation of Depth-Ambiguous Biological Motion Figures

**DOI:** 10.1371/journal.pone.0014725

**Published:** 2011-02-23

**Authors:** Ben Schouten, Nikolaus F. Troje, Jean Vroomen, Karl Verfaillie

**Affiliations:** 1 Laboratory of Experimental Psychology, Department of Psychology, Katholieke Universiteit Leuven, Leuven, Belgium; 2 Department of Psychology and School of Computing, Queen's University, Kingston, Ontario, Canada; 3 Department of Psychology, Tilburg University, Tilburg, The Netherlands; University of Minnesota, United States of America

## Abstract

**Background:**

The focus in the research on biological motion perception traditionally has been restricted to the visual modality. Recent neurophysiological and behavioural evidence, however, supports the idea that actions are not represented merely visually but rather audiovisually. The goal of the present study was to test whether the perceived in-depth orientation of depth-ambiguous point-light walkers (plws) is affected by the presentation of looming or receding sounds synchronized with the footsteps.

**Methodology/Principal Findings:**

In Experiment 1 orthographic frontal/back projections of plws were presented either without sound or with sounds of which the intensity level was rising (looming), falling (receding) or stationary. Despite instructions to ignore the sounds and to only report the visually perceived in-depth orientation, plws accompanied with looming sounds were more often judged to be facing the viewer whereas plws paired with receding sounds were more often judged to be facing away from the viewer. To test whether the effects observed in Experiment 1 act at a perceptual level rather than at the decisional level, in Experiment 2 observers perceptually compared orthographic plws without sound or paired with either looming or receding sounds to plws without sound but with perspective cues making them objectively either facing towards or facing away from the viewer. Judging whether either an orthographic plw or a plw with looming (receding) perspective cues is visually most looming becomes harder (easier) when the orthographic plw is paired with looming sounds.

**Conclusions/Significance:**

The present results suggest that looming and receding sounds alter the judgements of the in-depth orientation of depth-ambiguous point-light walkers. While looming sounds are demonstrated to act at a perceptual level and make plws look more looming, it remains a challenge for future research to clarify at what level in the processing hierarchy receding sounds affect how observers judge the in-depth perception of plws.

## Introduction

Adequately perceiving and understanding the actions of our conspecifics is a prerequisite for normal social development and interaction [1]. Since the findings of Johansson [2], point-light figures have proven to be very useful in studying the visual perception of actions or biological motion. Despite the limitation of the visual information to a small number of moving point-lights, observers are able to effortlessly judge a variety of behaviorally relevant properties. See Blake and Shiffrar [3] and Verfaillie [4] for reviews.

Although the specific neural correlates that subserve the visual perception of actions or biological motion remain unclear, a general consensus seems to exist on the involvement of two neural structures. First, the superior temporal sulcus (STS) has been reported in almost all neurophysiological studies on the perception of biological motion [e.g., 5,6,7]. Second, so-called mirror neurons were discovered in the premotor cortex (area F5) in monkeys [e.g., 8,9,10]. These neurons are not only active during action observation, but also when the monkey is performing the action. In humans, mirror neurons have been localized in the inferior frontal gyrus [Broca's area; e.g., 11]. The human mirror neuron system in premotor cortex has also been shown to respond to point-light biological motion [12], [13].

The focus in the research on biological motion perception traditionally is restricted to the visual modality. However, humans live in a multisensory world, and sounds are often available during action observation. Apart from speech - of which to date it is not clear whether it is a special case of audiovisual integration [14], [15], [16] - numerous actions provide observers with synchronized audiovisual stimulation, as in the case of walking, clapping, hammering, putting down a cup, etc. The perceptual system effortlessly integrates this multisensory stream of information. Therefore, it is sometimes hypothesized that actions are not represented merely visually but rather audiovisually.

Recent neurophysiological research supports this idea. First, audiovisual mirror neurons have been localized in the ventral premotor cortex of monkeys, neurons that not only fire when the monkey executes a particular action or sees the action, but also when the monkey hears the particular action related sound [17], [18]. Second, using fMRI in humans, Kaplan and Iacoboni [19] investigated the neural systems responding to the sight and sound of a bimanual paper tearing. Audiovisual facilitation was found in the ventral premotor cortex for the action stimuli but not for non-action control stimuli, suggesting that this region is involved in integrating multimodal information about actions. Third, with TMS Alaerts, Swinnen, and Wenderoth [20] explored responses of the human primary motor cortex (M1) to visual and auditory stimuli. They observed a selective response increase to congruent audiovisual manual actions, as compared to unimodal and incongruent multimodal manual actions. Fourth, Bidet-Caulet, Voisin, Bertrand, and Fonlupt [21] report that listening to the footsteps of a walker activates the posterior STS, a region that is, as mentioned before, active during the visual perception of biological motion. Finally, the data of Barraclough, Xiao, Baker, Oram, and Perret [22] support the hypothesis that STS is involved in the integration of audiovisual information about actions.

Inspired by the above described observations, several research groups have recently started to investigate the audiovisual perception of biological motion. For instance, Brooks, van der Zwan, Billard, Petreska, Clarke, and Blanke [23] reported, unlike data from previous studies investigating audiovisual integration in linear motion processing [24], [25], a left-right direction selective enhancement of the detection of a visually masked point-light walker. Relative to a control condition (stationary sound), auditory motion in the same direction as the visually defined biological motion target increased its detectability. Auditory motion in the opposite direction had the inverse effect. Second, using visually masked point-light displays of tap dancing feet and congruent synchronized sounds, Arrighi, Marini, and Burr [26] demonstrated audiovisual enhancement better than the optimal maximum likelihood prediction. Another group of researchers [27] designed a task in which observers had to decide whether a periodically moving point-light walker had the same temporal frequency as a series of auditory beeps that in some cases coincided with the footsteps of the walker. Performance in this task was consistently better for upright point-light walkers compared to inverted or scrambled walkers. Finally, van der Zwan, MacHatch, Kozlowski, Troje, Blanke, and Brooks [28] paired point-light walkers signalling gender on a continuum from extremely female to extremely male [29] with auditory footsteps that were perceived to be unambiguously female. Figures paired with female footsteps were judged more female. Moreover, adaptation to a gender-neutral walker paired with the female footsteps elicited male after-effects, indicating that the effects operate at the perceptual level.

The goal of the current study was to explore whether sound also affects the visual in-depth perception of point-light figures. Recently, the visual perception of depth-ambiguous biological motion attracted scientific interest [30]–[38]. An orthographic 2D projection of a symmetrical point-light walker is perceptually bistable: It is either perceived to be ‘facing the viewer’ (FTV) or it is perceived as ‘facing away’ from the viewer (FA). Nevertheless, observers exhibit a ‘facing bias’, that is, a perceptual bias to interpret the figure as being oriented *towards* the viewer [35].

## Results and Discussion

### Experiment 1

In Experiment 1, we examined whether auditory information that was congruent either with an approaching or with a receding walker would influence the in-depth interpretation of a depth-ambiguous point-light walker (plw). To this end, we presented to observers orthographically projected point-light walkers (plws) in four sound conditions. Walkers were either presented unimodally or they were paired with sounds synchronized to the footsteps. Sounds were either stationary or looming or receding. To induce apparent sound-depth [39] we increased/decreased the intensity level of subsequent tones to create looming/receding sounds, respectively [40]. Stationary sounds had a constant intensity level. On each trial, observers indicated whether they perceived the plw as facing towards them or facing away from them.

Plws usually elicit a strong facing bias. Therefore, to avoid ceiling effects in the looming condition, here we included, besides a plw that consistently elicits a facing bias, also a plw that consistently is perceived as rather ambiguous concerning its in-depth orientation (see methods). To minimize demand effects, observers were instructed to ignore the sound and to only respond to the visual information. Thus, if sounds had no effect, proportions of FTV responses should be similar in all sound conditions. Alternatively, if looming and receding sounds had an effect such that the perceived in-depth orientation was modified in the direction suggested by the sound, proportions of FTV responses should be higher in the looming condition and lower in the receding condition, compared to the no-sound condition. The proportions of FTV responses in the stationary condition should not differ from the no-sound condition because stationary sounds do not induce a particular in-depth interpretation.

**Figure 1:**
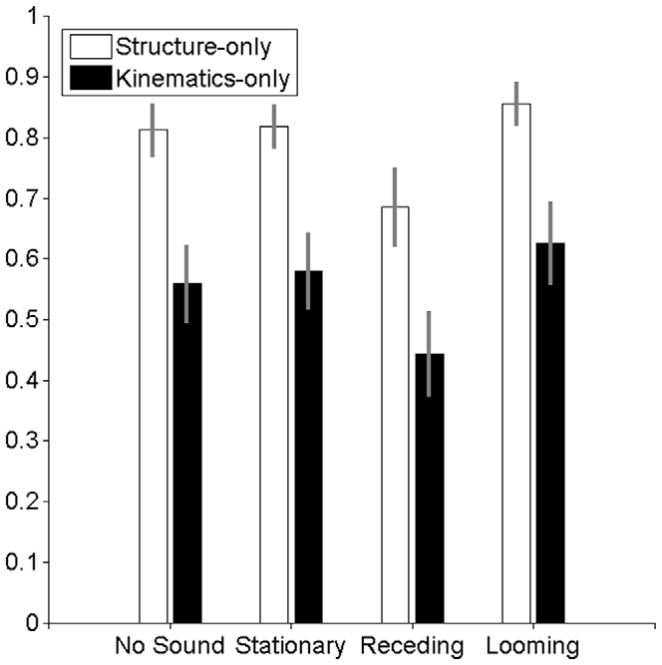
Proportions of FTV responses for both types of point-light figures and all sound conditions in Experiment 1. Mean proportions (across observers) of FTV responses for the structure-only male plws (white) and the kinematics-only male plws (black) in the no-sound, stationary, receding, and looming conditions. Error bars indicate +- 1 SE from the mean. Proportions of FTV responses are higher in the looming condition and lower in the receding condition compared to the no-sound and stationary condition. The effect of sound is similar for both types of point-light figures.

Figure 1 depicts the proportions of FTV responses for the two types of walkers in each of the four sound conditions. We analysed the data using a repeated measurement ANOVA and paired t-tests on probit transformed proportions. The average amount of FTV responses was 67%. Consistent with previous research [30], [33]–[38], we found a facing bias for plws. Also in line with previous research [38], there was a strong facing bias for the structure-only male plw in the no-sound condition (81% of FTV responses). This bias was significantly smaller for the kinematics-only male plw (56%, *t*(18) = −4.39, *p*<0.001). Also across all sound conditions, the number of FTV responses was smaller for the kinematics-only male plw (55%) than for the structure-only male plw (79%). This was confirmed by a repeated measurement ANOVA with plw type and sound condition as factors. There was a main effect of plw type (*F*(1,18) = 22.25, *p*<0.001). This analysis also revealed a main effect of sound condition (*F*(3,54) = 7.47, *p*<0.001). There was no interaction between sound condition and plw type (*F*(3,54) = 0.34, *p* = 0.796).

The crucial question in Experiment 1 was whether the amount of FTV responses in the looming condition was higher and in the receding condition was lower compared to the no-sound condition and the stationary condition. The amount of FTV responses in the stationary condition and in the no-sound condition should not differ. To test this, we performed five paired t-tests with a familywise alfa of 0.05 (all t-tests were two-sided). The Bonferroni corrected alfa for each test was 0.01. The results showed that the number of FTV responses in the looming condition (74%) was significantly higher compared to the no-sound condition (69%; *t*(37) = 3.33, *p* = 0.002), and compared to the stationary condition (70%; *t*(37) = 3.23, *p* = 0.003). The number of FTV responses in the receding condition (56%) was significantly lower compared to the no-sound condition (*t*(37) = −3.10, *p* = 0.004) and compared to the stationary condition (*t*(37) = −3.36, *p* = 0.002). The amount of FTV responses in the stationary condition and in the no-sound condition did not differ (*t*(37) = 0.04, *p* = 0.96). Overall, these results indicate that, despite the instruction to ignore the sound, the visually perceived in-depth orientation of plws was reliably shifted in the direction suggested by the sound. Sound affected plws that elicit a facing bias and plws that are perceived as more depth-ambiguous, in the same way.

Results show that looming sounds lead to more ‘facing the viewer’ responses whereas receding sounds have the opposite effect and lead to more ‘facing away’ responses. It seems that relative to the no-sound condition, receding sounds produced a somewhat stronger modulation effect (about 13% change) as compared to looming sounds (about 5% change). This is probably due to a ceiling effect in the looming sound condition. Indeed, if we consider all 38 proportions of FTV responses (19 observers x 2 types of point-light walkers) in the looming sound condition, 16 (42%) of those proportions exceed .90 and nine (24%) of those proportions exceed .95 suggesting (despite the inclusion of the perceptually depth-ambiguous kinematics-only male plw) a ceiling effect. The size of the effect of looming sound on the perceived in-depth orientation found in Experiment 1 may thus be an underestimation.

The current data suggest that looming and receding sounds affect the in-depth interpretation of plws. Despite the instruction to ignore the sound and to only report the visually perceived in-depth orientation, observers' judgements were affected by looming and receding sounds. It may thus be possible that, in accordance with recent studies documenting on changes in the phenomenological experience of biological motion stimuli when a congruent sound is available [23], [26]–[28], here, in the looming and receding conditions, audiovisual integration led to phenomenological different perceptual experiences than in the stationary and the no-sound condition. After completion of the experiment but before debriefing, participants were asked some questions. One of the questions was to estimate how many *visually distinguishable* plws were presented during the experiment. We expected participants to be able to answer this question quite accurately, because only two easily distinguishable types of plws were used. Indeed, all participants noticed there were at least two different plws. However, only 21% of the participants (4/19) gave the correct response (two) whereas the majority, 63% of the participants (12/19), indicated that they had seen three or four different plws (spontaneous responses were ‘three’, ‘four’, or ‘three or four’). The rest of the observers (16% or 3/19) thought there were even more than four visually distinguishable plws. Although memory reports should always be interpreted with care, the observation that 79% of the participants spontaneously responded to have seen more than two visually distinguishable plws, may suggest that also in the current experiment the simultaneous presentation of sound led to phenomenological different perceptual experiences of the plws than when they were presented without sound. However, based on the present data we cannot exclude the possibility that the observed effects are the result of response bias. This was the rationale for designing Experiment 2.

### Experiment 2

The goal of Experiment 2 was to test whether the effect observed in Experiment 1 operates at a perceptual level, that is, to test whether looming or receding sounds can change the visual phenomenological experience of a plw rather than only drive response bias. In Experiment 1, only orthographically projected plws were presented and subjective responses on the perceived in-depth orientation were recorded. As orthographic plws contain no objective cues as to their in-depth orientation there was, in fact, no right or wrong response. The type of task in Experiment 1 did thus not allow to test for changes in performance.

One way to test whether looming and receding sounds affect visual *perception* is by examining whether the addition of looming or receding sounds to an orthographic plw changes task performance when this audiovisual plw has to be visually compared directly to another plw that carries visual cues making it objectively either facing towards or facing away from the viewer.

Schouten and Verfaillie [34] recently developed a technique to gradually manipulate the perceived in-depth orientation of plws with perspective cues. In Experiment 2, we employed this technique to create, in addition to the orthographic plws, plws that were, because of the added perspective information, either objectively facing the viewer or facing away from the viewer. More specifically, we set up a temporal 2AFC task in which observers perceptually compared the two types of plws. In one interval (first interval in half of all trials; second interval in the other half) a plw was presented with perspective cues that made the stimulus either facing toward (looming) or facing away (receding) the observer. In the other interval an orthographically projected and therefore theoretically depth-ambiguous plw was presented without sound or paired with either looming or receding sounds. On each trial observers indicated the interval (first or second) that contained the plw that was *visually* most convincingly perceived as facing the viewer. For example, in the trials in which the first interval contained the orthographically projected plw and the second interval contained a looming plw, observers had to indicate the second interval, but when the second interval contained a receding plw they had to indicate the first interval. Feedback (correct - incorrect) was provided after each response. Observers were instructed to ignore the sound and to use the feedback after each trial to optimize their task performance.

This setup allows for stimulus comparisons at the perceptual level and a measurement of changes in perceptual experiences by examining increases or decreases in task performance. Indeed, if sound has no perceptual effect on the visual perception of plws, performance in the 2AFC task should be independent of whether the orthographic plw is presented without sound or paired with a looming or receding sound. Alternatively, if the addition of sound to the orthographic plw has a perceptual effect that cannot be ignored, then in particular conditions in the present design sound should improve performance while in other conditions it should decrease performance. For example, take a first type of trial in which one interval contains a looming plw and the other interval an orthographic plw that is paired with a receding sound. Imagine also a second type of trial that is identical to the first type of trial except that in the interval with the orthographic plw no sound is presented. When a receding sound has a perceptual effect in the sense that it makes the orthographic plw look more convincingly receding or less looming then it could be expected that performance (the ability to correctly indicate the interval with the plw that is most looming) in the first type of trial is better than in the second type of trial. Alternatively, if in the second type of trial the interval with the orthographic plw is paired with a looming sound then it could be expected that performance in the second type of trial is worse than in the first type of trial because the looming sound makes the orthographic plw look more like the looming plw.

Performance changes in this task that are expected when the sounds have a perceptual effect on how the plws are perceived can be tested by computing whether differences in performance scores between particular conditions turn out to be positive. Under the hypothesis that a looming sound has an effect that cannot be ignored on how the plw is perceived, we expect differences between performance scores in the looming sound condition and the no-sound condition (orthographic walkers) for trials containing receding walkers (perspective walkers) and differences between the no sound condition and the looming sound condition (orthographic walkers) for trials containing the looming walkers (perspective walkers) to be positive. Likewise, under the hypothesis that a receding sound has a perceptual effect that cannot be ignored, the difference between performance in the no-sound condition and the receding sound condition (orthographic walkers) for trials containing receding walkers (perspective walkers) and the difference between performance in the receding sound condition and the no-sound condition (orthographic walkers) for trials containing the looming walkers (perspective walkers) is expected to be positive.

**Figure 2:**
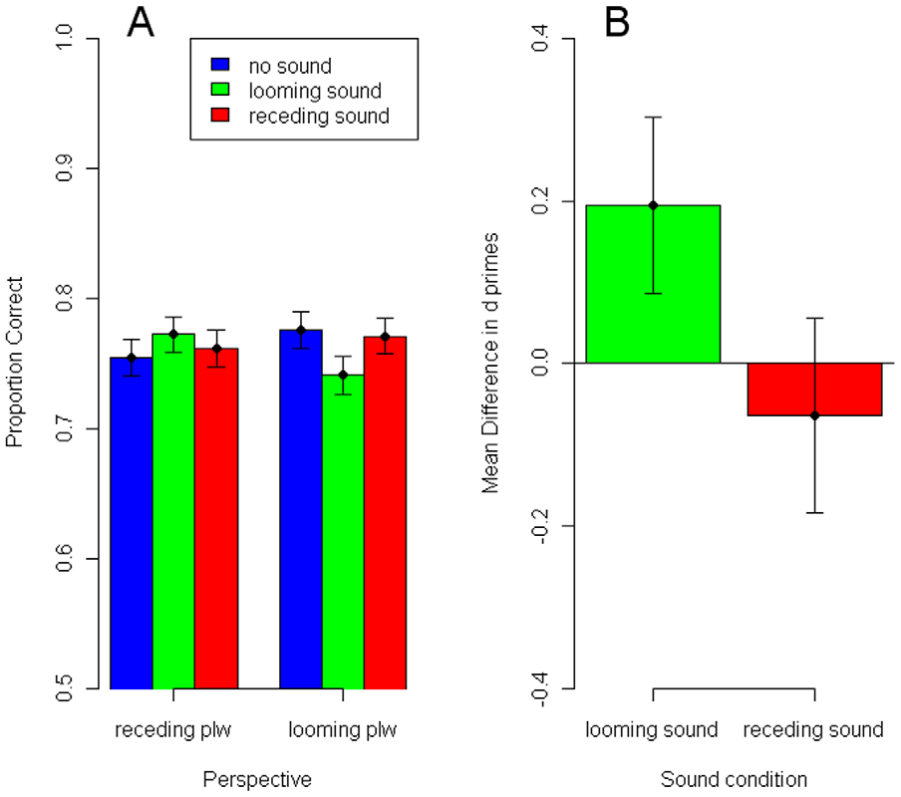
Results of Experiment 2. Mean proportions correct and mean difference scores. A. Mean proportions (across observers) of correct responses for each sound condition and for trials with receding plws and trials with looming plws. Compared to the no-sound condition (blue bars) in the looming sound condition (green bars) performance increased in trails with receding plws and decreased in trials with looming plws. In the receding sound condition (red bars), performance did not differ from the no-sound condition (blue bars). B. Mean of the differences in d-primes between the looming sound condition and the no-sound condition for trials with receding plws and between the no-sound condition and the looming sound condition for trials with looming plws (green bar) and mean of the differences in d-primes between the no-sound condition and the receding sound condition for trials with receding plws and between the receding sound condition and the no-sound condition for trials with looming plws (red bar). Difference scores for the looming sounds were positive indicating that looming sounds make an orthographic plw look more looming. Difference scores for receding sounds were not positive. Error bars in A and B indicate +- 1SE from the mean.

In Figure 2A we plot the mean proportions of correct responses for each of the three sound conditions (no sound = blue; looming sound = green; receding sound = red) and for trials containing receding (left) and looming (right) plws (the three levels of looming perspective were averaged and the three levels of receding perspective were averaged). First, under the hypothesis that looming sounds (green bars) affect the perceived in-depth orientation of orthographic plws, relative to trials without sound (blue bars), we should observe an increase in performance for trials containing receding plws and a decrease in performance for trials containing looming plws. This is exactly what we observe in Figure 2A. For trials with receding plws, relative to the no-sound condition (75,44%), performance is better in the looming sound condition (77,22%). However, for trials with looming plws, relative to the no-sound condition (77,56%) performance is worse in the looming sound condition (74,11%). Second, under the hypothesis that receding sounds (red bars) affect the perceived in-depth orientation of orthographic plws, relative to trials without sound (blue bars) we should observe a decrease in performance for trials containing receding walkers and an increase in performance for trials containing looming walkers. However, this is not what we observe in Figure 2A. The receding sound condition does not seem to differ much from the no-sound condition (76,11% vs. 75,44% for the receding walkers and 77,11% vs 77.55% for the looming walkers, respectively).

These observations are confirmed by statistics. For each observer, d-primes were computed for each of the six combinations of direction of perspective cue (receding plw vs. looming plw) and sound condition (no sound, looming sound, receding sound for the orthographic plw). Second interval responses in trials in which the most looming plw appeared in the second interval (signal trials) were coded as hits. Second interval responses in trials in which the most looming plw was presented in the first interval (noise trials) were coded as false alarms. To test the effect of looming sounds, differences were computed between the d-primes in the looming sound condition and the no-sound condition for trials with receding plws and between the d-primes in the no-sound condition and the looming sound condition for trials with looming plws (see green bar Figure 2B). If looming sounds indeed make plws look more looming then these difference scores are expected to be significantly positive. A one-sided t-test revealed that this was the case (*t*(49) = 1.80, *p* = 0.039). To test the effect of receding sounds, differences were computed between d-primes in the no-sound condition and the receding sound condition for trials with receding plws and between d-primes in the receding sound condition and the no-sound condition for trials with looming plws. As suggested by Figure 2B (red bar) these difference scores were not found to be significantly positive (*t*(49) = −0.53, *p* = 0.702).

The results of Experiment 2 show significant changes in task performance when looming sounds are paired with orthographic plws. The changes in task performance that are observed are exactly the changes that would be expected if looming sounds have a perceptual effect on the visually perceived in-depth orientation. When observers have to judge whether either an orthographic plw or a plw with looming perspective cues is visually most looming it becomes harder when the orthographic plw is paired with looming sounds. When observers have to judge whether either an orthographic plw or a plw with receding perspective cues is visually most looming it becomes easier when the orthographic plw is paired with looming sounds. These observations show that looming sounds make an orthographic plw look more looming.

We also tested whether a receding sound leads to changes in performance. However, we did not find any significant changes in task performance when receding sounds were added to the orthographic plw. Potential reasons for this apparent asymmetry will be further discussed in the General Discussion.

### General Discussion

The goal of the present study was to test whether looming and receding sounds affect the perceived in-depth orientation of depth-ambiguous plws. Results of Experiment 1 showed that observes judge orthographic plws paired with looming sounds as more looming and orthographic plws paired with receding sounds as more receding compared to orthographic plws paired with stationary sounds or presented without sound. However, despite the fact that observers in Experiment 1 were instructed to ignore the sound, one could argue that the results of Experiment 1 do not necessarily reflect a perceptual effect. The observed changes in responses could be due to response bias. In Experiment 2 we eliminated the potential effect of response bias. Results showed that in a task in which on each trial an orthographic plw was compared with a plw containing perspective cues that made it objectively either facing the viewer or facing away, the addition of looming sounds to the orthographic plw led to significant changes in task performance indicating that the effect of looming sounds acts at the perceptual level. However, in Experiment 2 we did not observe any performance changes when a receding sound was added to the orthographic plw. Hence, while observers were unable to ignore looming sounds, they were able to ignore receding sounds.

A possible explanation of the absence of an effect of receding sounds in Experiment 2 is that receding sounds, under particular circumstances, affect the perceived in-depth orientation of plws but that in Experiment 2 it was easier to ignore receding sounds than to ignore looming sounds. For example, one reason why receding sounds might have been more easily ignored in Experiment 2 could be related to the type of plw to which the sounds were paired, namely the orthographic plw. As demonstrated by many studies before [30], [33]–[38] this type of plw is, despite the absence of any objective cues to depth, actually perceived as facing the viewer in about 70% to 80% of the cases. This visual phenomenon, known as the facing bias might to some extent have prevented receding sounds to bind to the plw. For instance, when the perceptual system detects incongruence between the visual and auditory information it might ignore the one or the other. Note that in Experiment 2 the task instruction was to ignore the sound and to make a visual comparison between the sequentially presented plws. It is thus possible that observers were better able to ignore receding sounds because they were always paired with a plw that was perceived as facing the viewer in most of the cases and therefore incongruent with the sound. In that respect looming sounds might have had a benefit compared to receding sounds because in most of the cases they were congruent with the visual information.

Another reason why receding sounds are easier to ignore than looming sounds might be that receding sounds are behaviorally and socially less relevant than looming sounds. In fact, Neuhoff [40] demonstrated a perceptual bias for rising tones. Listeners reliably overestimated the change in level of rising level tones relative to equivalent falling level tones. According to Neuhoff, in a natural environment this overestimation could provide a selective advantage because rising intensity can signal movement of the source towards an organism (also see [41]). Note that likewise the facing bias for orthographic plws has been hypothesized to reflect evolved perceptual biases [30], [33]. It is thus possible that the perceptual system tends to give a higher weight to information signalling movement of a source towards the organism and is therefore more unwilling to ignore looming sounds and more willing to ignore receding sounds.

To what extent are the effects observed in the present study specific to biological motion? Kitagawa and Ichahara [42], for example, demonstrated an auditory aftereffect from adaptation to visual (non-biological) motion in depth. After a few minutes of viewing a square moving in depth, a steady sound was perceived as changing loudness in the opposite direction. Interestingly, however, listening to a sound changing in intensity did not affect the visual changing-size aftereffect. Kitagawa and Ichahara [42] did thus not observe an effect of sound on vision for non-biological motion stimuli whereas in the present study we observed an effect of looming sounds on the visual perception of biological motion. While this could indicate that biological motion is necessary to observe an effect of sound on the in-depth perception of visual stimuli, the present data do not allow to draw conclusions on this issue. Ideally, it could be tested, for example, whether looming sounds also affect inverted plws. Inversion of the plw is an often used procedure to provide a control condition with non-biological motion because an inverted plw contains equivalent low-level stimulus properties as an upright plw [43]. However, pilot experiments in our lab show that observers perform near chance-level when having to indicate the in-depth orientation of an inverted plw that is disambiguated with perspective cues. Note also that the rhythmic sounds accompanying the plws in the present study are not real recorded footsteps but synthetically generated sounds that either rise or fall in intensity. The sounds are only meaningfully interpreted as ‘footsteps’ because they are paired with a swarm of dots that is interpreted as a walking person (which is not the case for inverted plws). In conclusion, it is far from trivial to design a fair control condition that allows to test whether the present effect is specific to biological motion. In other words, it is hard to design a stimulus that, in terms of its low-level stimulus properties, is close enough to the meaningful audiovisual biological motion stimulus but yet different enough from it to be not biologically meaningful.

If the effects of looming sounds observed in the present study were to be specific to biological motion then the neural structures most likely to be involved might be the structures containing multisensory neurons specifically tuned to the perception and/or production of specific action patterns, namely audiovisual mirror neurons [17],[18]. STS would also be a likely candidate. However, the involvement of STS might not be specific to biological motion as it has also been shown to be involved in audiovisual integration of non-biological motion stimuli [44].

Finally, we like to note that Kitagawa and Ichahara [42] in their study found that adaptation to a combination of auditory and visual stimuli changing in a compatible direction increased the auditory aftereffect. However, when visual stimuli were incongruent with auditory stimuli the effect of audiovisual adaptation was about the same as the effect of auditory adaptation, suggesting that when audiovisual stimuli were incongruent the visual stimulus was ignored (also see [45]). This observation is consistent with the account assuming that receding sounds in our Experiment 2 were ignored because they were incongruent with the visual stimulus.

In summary, data of Experiment 1 indicated that, despite the instruction to ignore the sound, observers judged a plw paired with sounds rising in intensity - and thus suggesting looming motion - as more looming and a plw paired with sounds falling in intensity - and thus suggesting receding motion - as more receding compared to when no sound was available or when a stationary sound was available. Results of Experiment 2 demonstrated that the effect of looming sounds on how the visual appearance of a plw is judged acts at a perceptual level. That is, looming sounds make plws look more looming. It remains a challenge for future research to examine whether the effect of looming sounds observed in the present study is specific to biological motion. Future research might also clarify at what level in the processing hierarchy receding sounds affect how observers judge the in-depth perception of plws.

## Methods

### Experiment 1

#### Participants

Nineteen students from the University of Leuven participated for course credit. All observers had normal or corrected to normal vision and hearing and were naïve to the purpose of the experiment.

#### Ethics Statement

Written informed consent was obtained prior to the experiment. The study was approved by the Ethical Committee of the Faculty of Psychology and Educational Sciences of the University of Leuven and in accordance with the ethical standards laid down in the 1964 Declaration of Helsinki.

#### Stimuli and apparatus

There were two types of plws: A structure-only and a kinematics-only male plw both derived from a gender continuum. A structure-only male plw was composed of gender neutral kinematics (z-score: 0 std) and male structure (6 std). A kinematics-only male plw was composed of gender neutral structure (0 std) and male kinematics (6 std). See Troje [29], [46] for details on stimulus creation. Note that in the present study the gender of the plw is irrelevant. In Schouten, Troje, and Verfaillie [38] it was shown that changes in the facing bias as a function of figure gender (see [30], [33]) are not caused by changes in perceived gender but result from structural and kinematic stimulus properties. Here, we simply employ the empirical finding that particular stimulus properties consistently elicit a particular proportion of FTV responses. The choice of these two types of plws in the present study is thus solely based on their earlier observed property to either elicit a facing bias (the structure-only male plw; about 81% of FTV responses) or to elicit a rather ambiguous in-depth interpretation (the kinematics-only male plw; about 54% of FTV responses). Each plw was orthographically projected in frontal view in the centre of the screen (subtending a size of 8° of VA height) and each consisted of 15 black dots (radius = 15 arc mins of VA) on a grey background. Total trial duration always matched the duration of 4 walking cycles, that is, 8 steps (96 frames per cycle at 85 fps = 1130 ms per cycle; 384 frames in total = 4518 ms). Visually, trials consisted of a grey background presented for a duration of 3 step cycles (288 frames = 3388 ms). Then, the plw appeared for one step cycle (two steps; 96 frames = 1130 ms). This was done to prevent observers to only rely on visual information near trial onset. The sound was played during the entire trial and a convincing looming or receding auditory percept was only obtained near the end of the trial. Auditory stimuli representing the footsteps consisted of eight 30 ms sine waves of 200 Hz, amplitude modulated depending on the required sound level. To avoid clicks, the first 15 ms of the sine waves were ramped. Note that the objective point of audiovisual simultaneity for these plws is unknown because the sounds of the footsteps were not recorded during the motion capture phase of the stimulus creation [29]. Audiovisual synchrony was therefore set to match the subjective point of simultaneity as obtained in an earlier adjustment task (see e.g. Schouten & Verfaillie, 2006, *The audiovisual perception of biological motion*, Perception 35, ECVP Abstract). The adjustment task provided the frame of the step cycle to which the sound had to be coupled to obtain maximum perceived synchrony. This point was then set to represent an audiovisual offset of 0 ms. In the looming condition the sounds linearly increased in intensity from 40 dB to 64 dB SPL. To mimic the decrease in auditory delay of an approaching sound source due to the slow travel time of sound (approximately 343 m/s), for each step (approximately 70 cm in natural circumstances) the audiovisual offset was decreased with 2 ms (from 14 ms to 0 ms; positive values indicate auditory delay). The reverse settings (linearly decreasing intensity from 64 dB to 40 dB SPL and linearly increasing audiovisual offset from 0 ms to 14 ms) were applied to create receding sounds. In the stationary condition the sound level (55 dB SPL) and the audiovisual offset (0 ms) were constant. In the no-sound condition no sound was presented. On each trial the start posture of the plw was randomly chosen from the first 24 frames of the step cycle.

#### Procedure

All observers were tested individually in a dimly lit and sound attenuated room, sitting in front of a 21 inch CRT monitor at a viewing distance of 57 cm. Sounds were presented by three Dell speakers located 80 cm behind the screen: two satellite speakers (distance between left and right speaker was 70 cm) and one subwoofer, located centrally. Observers were asked to indicate on each trial by a key-press whether the plw was perceived as facing towards them or facing away from them (arrow down for FTV, arrow up for FA). Observers were explicitly instructed to ignore the sound and to only report the visually perceived in-depth orientation. It was stressed that an equal distribution of both response alternatives was not necessary. After instructions, observers completed 32 practice trials (random selection of all possible conditions). Thereafter, the experiment commenced. Trials of all conditions were randomized and then divided in blocks of 32 trials between which participants could take a break. In total each participant completed 320 trials (2 types of plws * 4 sound conditions * 40 repetitions).

### Experiment 2

#### Participants

Twenty-five students from the University of Leuven participated for course credit. All observers had normal or corrected to normal vision and hearing and were naïve to the purpose of the experiment.

#### Ethics Statement

Written informed consent was obtained prior to the experiment. The study was approved by the Ethical Committee of the Faculty of Psychology and Educational Sciences of the University of Leuven and in accordance with the ethical standards laid down in the 1964 Declaration of Helsinki.

#### Stimuli and apparatus

Computer screen and speaker setup was the same as in Experiment 1. As in Experiment 1 orthographic plws were created by orthographically projecting the 3D coordinates created by Troje [29]. However, in Experiment 2, only the coordinates of the gender neutral plw (z-score: 0 std) were used. Using the technique described in detail by Schouten and Verfaillie [34] using different levels of perspective three plws were created that were objectively oriented towards the observer and three plws were created that were objectively oriented away from the viewer. Distance manipulations were 3, 6, and 12 times the height of the walker at both the front and the back of the walker resulting in field of view angles of 67°, 37°, and 19° at both sides. The height of the point-light figures subtended about seven degrees of visual angle. Each of the 15 dots of the plw subtended about 15 arc mins. The start posture of the animation cycle (117 frames/cycle) was randomized across trials. Auditory stimuli and their relative synchronisation to the orthographic plws were similar as in Experiment 1, except that in Experiment 2 only looming and receding sounds were used and that orthographic plws were visible during the entire presentation of the sound (4 walking cycles).

#### Procedure

In Experiment 2, on each trial observers were presented with two sequential intervals. Each trial contained an orthographic plw and a plw with added perspective cues to make it either objectively facing towards (half of the trials) or facing away from the observer (other half of the trials). Walkers were walking as if on a treadmill. Presentation order (orthographic plw in first interval and plw containing perspective in second interval or vice versa) was counter balanced. Each interval started with a 500 ms fixation cross followed by the presentation of the plw in the centre of the screen during 3900 ms (four walking cycles of 975 ms). The time between the disappearance of the first plw and the fixation cross of the next interval was 500 ms. After the disappearance of the second plw observers indicated either the first (response key 1) or the second (response key 2) interval, initiating the next trial after a 500 ms blank. To illustrate the task, before the start of the experiment observers were shown four example trials (random selection). Observers were instructed to indicate the interval containing the plw that was most convincingly looming. It was clarified that when the plws in both intervals were perceived as facing away they should indicate the plw that was perceived as less convincing facing away. Observers were instructed to ignore the sound and to make a visual comparison between the stimuli in both intervals. After checking whether the instructions were clear observers completed a practice block of 27 trials (random selection of all possible trials). After the practice block each observer completed 216 trials (three perspective levels * two facing directions * three sound conditions * two intervals * six repetitions) divided in eight blocks of 27 trials (order of trials was randomized). Between blocks observers were allowed to take a short break. In total the experiment lasted almost one hour.
